# Thermally Driven Flow of Water in Partially Heated Tall Vertical Concentric Annulus

**DOI:** 10.3390/e22101189

**Published:** 2020-10-21

**Authors:** Jawed Mustafa, Saeed Alqaed, Mohammad Altamush Siddiqui

**Affiliations:** 1 Mechanical Engineering Department, College of Engineering, Najran University, Najran 61441, Saudi Arabia; saalqaed@nu.edu.sa; 2 Department of Mechanical Engineering, Z.H. College of Engineering & Technology, Aligarh Muslim University, Aligarh 202001, India; maltamushsiddiqui@gmail.com or

**Keywords:** natural convection, tall vertical annulus, CFD, modelling, heat transfer, fluid flow, buoyancy induced, partially heated

## Abstract

Computational fluid dynamics (CFD) has become effective and crucial to several applications in science and engineering. The dynamic behavior of buoyancy induced flow of water in partially heated tall open-ended vertical annulus is analyzed based on a CFD technique. For a vertical annulus, the natural convective heat transfer has a broad application in engineering. The annulus is the most common structure used in various heat transmission systems, from the basic heat transfer device to the most sophisticated atomic reactors. The annular test sections of such a large aspect ratio are of practical importance in the design of equipment’s associated with the reactor systems. However, depending on the geometrical structure and heating conditions, it exhibits different flow behavior. The annulus may either be closed or open-ended. In this study, we carry out CFD analysis to examine the thermodynamics properties and the detailed thermal induced flow behavior of the water in Tall open-ended vertical concentric annuli. The purpose of this study is to evaluate the impact of a partially heating on mechanical properties and design parameters like Nusselt number, mass flow rate and pressure defect. For Rayleigh number ranging from 4.4 × 10^3^ to 6.6 × 10^4^, while the Prandtl number is 6.43, the numerical solution was obtained. The modelling result showing the measurement and transient behavior of different parameters is presented. The numerical results would be both qualitatively and quantitatively validated. The presentation of unstable state profiles and heat variables along the annulus are also discussed.

## 1. Introduction

The process of energy transfer on a surface to a fluid flowing over it; as a result, the difference between them is referred to as convection heat transfer. Since the rate of convective heat transfer is influenced by the flow field of the fluid, it will strongly depend upon how the flow is generated. For forced convection, the flow of fluid is due to some external means such as fan or pump. While in free convection, the body forces are responsible for the flow and that occur due to the density difference arising caused by the changes of temperature in the flow field. The body forces are generated due to pressure gradients imposed on the whole fluid due to gravity. This results in the buoyancy force that causes the lighter fluid to rise upward. Therefore, the buoyancy induced flow is generally due to density gradient in the flow, which can also occur due to concentration gradient and temperature gradient. Thermo-siphon action is induced either in the cavity or in closed loop geometry due to heating and cooling of a fluid differentially. Energy systems (solar collectors), wall with multi-layers, windows with double glasses, in nuclear reactors, furnaces of distillery plant and several heat exchangers is also possessing thermally induced flow. Some practical structures like tubes of circular, annular cavities and parallel plates often require convective transfer of heat, mass flow in heated vertical open-ended annuli. The practical importance of such system are particularly the fuel elements of nuclear reactors, arrangements of double pipes, process of chemical distillery, solar energy collector for channel type, thermo protection system, electrical cables (gas cooled). Significant interest has been shown in these problems with natural convective heat transfer in recent years.

Thomas [1] analyzed numerically the fluid flow in an annular cavity closed from both ends, created by concentric two cylinders vertically and horizontally two planes. The analysis was Rayleigh number (Ra) up to 2 × 10^5^, 0.5 < Pr < 5, radius ratio from 1 to 4 and aspect ratio varies from 1 to 20. The motion was found to be produces by the gradient of radial density induced by thermal boundary conditions, maintaining the internal cylinder heated and the outer cylinder cold. The motion also consists of a single cell with low Rayleigh numbers, while a multicellular motion on large Rayleigh numbers can be observed. Mochimaru [2] developed a way to improve the solution of the transient natural convective transfer of heat in enclosures. The equation of motion, energy and continuity can also be differentiated in the Fourier series using the additional trigonometric function formulas, reducing the variables and lowering the calculation time. Ho and Lin [3] carried out numerical experiments with finite difference methods on the natural convective transfer of heat for cold water. Numerical results were reached for the radius ratio of 2.6 with a Rayleigh number ranging from 10^3^ to 10^5^. The inversion parameters were 0 to 1, the eccentricity was 0 to 0.8 and the cylinder orientation angle was 0 to π. The results indicates that the characteristics of transfer of heat and flow pattern are mostly affected by a combined effect caused by the water density inversion and the inner annulus cylinder position. El-Shaarawi and Al-Attas [4] investigate numerically, the behavior of laminar free convective transfer of heat in a vertical annulus with time for 4 < modified Gr < 50,000 and Pr = 0.7. At the small-time values, the temperature overshoot phenomena, that is, because of the superiority of conduction over the mode of convective transfer of heat, has been found to be more pronounced as the dimensionless annulus height decreases, that is, modified Gr when increases. El-Shaarawi et al. [5] investigated annulus geometric parameters and their effect on transfer of heat and rate of induced flow. The numerical results were provided with Pr = 0.7, showing that the geometry parameters like radius ratio and eccentricity had a significant impact on the results. Sankar and Younghae [6] studied the impact of a separate heating on convective transfer of heat for cylindrical annuli. During their study, the inner cylinder of the annulus had two separate source of heat like flush-mounted and the outer cylinder was maintaining at constant lesser temperature. The horizontal walls at bottom and top were kept adiabatic. The empirical tests reveal that at the bottom heater, the rate of heat transfer has always been higher, increasing as increase in radii ratio while decreasing as increase in the aspect ratio. Desrayaud et al. [7] conducted a comparative experiment to test the sensitivity of natural convection for four open boundary conditions in an asymmetric vertical heated channel and to define a benchmark solution for each of the boundary conditions. Their findings indicate that the return flow takes place at the outlet of channel and has also demonstrated that the change in flow patterns will not substantially change the flow rate of the outlet of the channel. The experiment and numerical analysis on a tall vertical open-ended concentric cylindrical annulus was also performed by Mustafa et al. [8,9,10]. Mohamad et al. [11] performed numerical investigation for the unsteady state, natural convection in the annular cylinders. Time needed for fully charging the storage tank and rate of heat transfer was calculated. It was found that a convection-operated storage tank reduces the thermal charging process time drastically compared with the thermally diffusion charging process. Lee et al. [12] conducted numerical analysis, in his research, unsteady three-dimensional incompressible Navier-Stokes equations are solved to simulate experiments. Unsteady time marching is proposed for a time sweeping analysis of various Rayleigh numbers. The accuracy of the natural convection data of a single horizontal circular tube can be guaranteed when the Rayleigh number based on the tube diameter exceeds 400. The aim of this work is to carry out a numerical study for detailed thermally induced behavior of water flow and also on the effect of partial heating in the open-ended Tall vertical annulus. Dynamic behavior of flow fluid is also analyzed for various fluxes of heats. In this study, the annuli had a radius ratio (outer radius to inner radius) equals to 1.184 and an aspect ratio (length to annular gap) of 352. Taken as a whole, we emphasize quantifying the impact of partial heating on design parameters like pressure distribution and coefficient of heat transfer for a very high aspect ratio and we further aim to identify flow physics as a flow pattern within the annuli.

## 2. Problem Formulation and Method of Solutions

**Figure 1:**
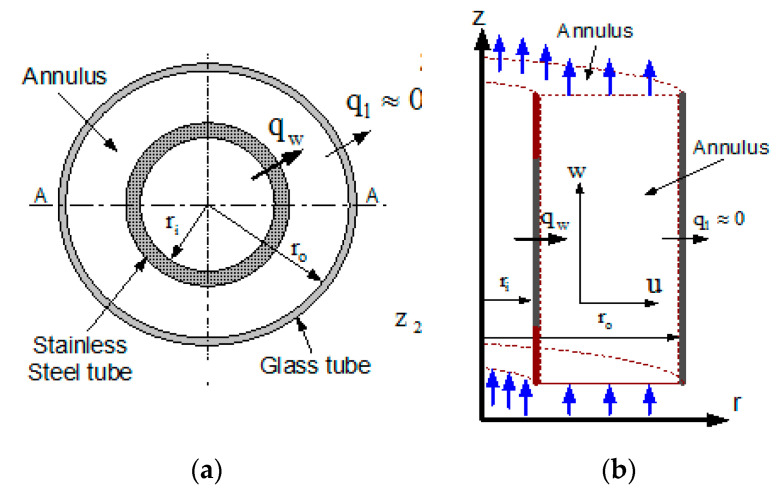
Schematic diagram (**a**) Plan of the thermo-siphon (**b**) computational geometry (not drawn to scale).

The schematic diagram of the problem for numerical analysis is shown in Figure 1, on which numerical computation was done. Figure 1a shows top view of the annulus indicating the various heat flux boundary condition while Figure 1b shows, the computational geometry of the problem. For the numerical simulation, two cases were taken. In the first case, the inner wall of the annulus is fully heated, hereafter referred to as full heating (FH). In the second case, the inner wall of the annulus is partially heated, hereafter referred to as partial heating (PH). For both cases, the outer wall is taken adiabatic. It is assumed that the test liquid (water used in experimental setup) is entering from the bottom of the annulus and leaves from the top end of the annulus, as shown in Figure 1b. Numerical analysis was conducted using the axisymmetric flow using a cylindrical coordinate system. In addition, the assumption of Boussinesq was employed to analyze the fluid. Table 1 shows the variable used in the equation. By introducing the reference variable of the following quantities (length (b), Time ($b^{2}/\alpha )$, Velocity (α/b), Temperature ($\frac{\left|q_{w}\right|b}{\kappa })$ and pressure ($\left(\rho \right){\left(\frac{\alpha }{b}\right)}^{2})$. The governing equations are transformed as follows:

(1) $$R=\frac{r}{b},\;Z=\frac{z}{b},\;U=u\left(\frac{b}{\alpha }\right),\;W=w\left(\frac{b}{\alpha }\right),\;\tau =t\left(\frac{\alpha }{b^{2}}\right),\;P=p\left(\frac{1}{\rho }\right){\left(\frac{b}{\alpha }\right)}^{2},\;\theta =\frac{T-T_{a}}{\frac{\left|q_{w}\;\right|b}{\kappa }},\;A=\frac{L}{b},\;\;\mathrm{RR}=\;\frac{r_{o}}{r_{i}},\;\;\;\;\;\;\frac{1}{R}\frac{\partial \left(\mathrm{RU}\right)}{\partial R}+\frac{\partial W}{\partial Z}=0$$

(2) $$\frac{\partial U}{\partial \tau }+U\frac{\partial U}{\partial R}+W\frac{\partial U}{\partial Z}=-\frac{\partial P}{\partial R}+\mathrm{Pr}\left[\frac{1}{R}\frac{\partial }{\partial R}\left(R\frac{\partial U}{\partial R}\right)-\frac{U}{R^{2}}+\frac{\partial^{2}U}{\partial Z^{2}}\right]$$

(3) $$\frac{\partial W}{\partial \tau }+U\frac{\partial W}{\partial R}+W\frac{\partial W}{\partial Z}=-\frac{\partial P}{\partial Z}+\mathrm{Ra}\;\mathrm{Pr}\;\theta +\mathrm{Pr}\left[\frac{1}{R}\frac{\partial }{\partial R}\left(R\frac{\partial W}{\partial R}\right)+\frac{\partial^{2}W}{\partial Z^{2}}\right]$$

(4) $$\frac{\partial \theta }{\partial \tau }+U\frac{\partial \theta }{\partial R}+W\frac{\partial \theta }{\partial Z}=\frac{1}{R}\frac{\partial }{\partial R}\left(R\frac{\partial \theta }{\partial R}\right)+\frac{\partial }{\partial Z}\left(\frac{\partial \theta }{\partial Z}\right),$$

where the Prandtl number (Pr) $=\;\frac{\nu }{\alpha }$ and the Rayleigh number (Ra) $=\frac{g\beta \left|q_{w}\right|b^{4}}{k\nu \alpha }$ is expressing the strength of buoyancy.

**Figure 2:**
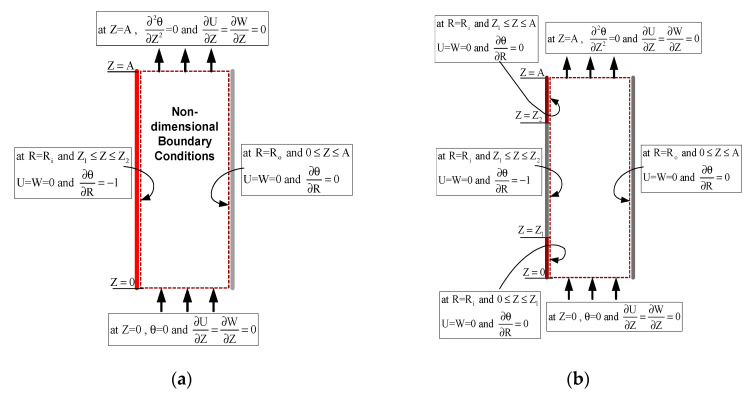
Non-Dimensional Boundary Conditions for (**a**) Full heating case (**b**) Partial heating case.

The above equations are solved simultaneously for the specified boundary conditions of fully heated and partially heated vertical concentric cylindrical annulus to see the natural convection phenomenon. The Figure 2a shows the non-dimensional form of boundary conditions for full heating case while Figure 2b shows for partial heating case of the geometry problem. For the partial heating case, the non-heating region of the inner cylinder at the inlet of annulus is Z = 0 to Z = Z_1_ = 21 (dimensionless height). From Z = Z_1_ = 21 to Z = Z_2_ = 300 is the heated region for inner cylinder while Z = Z_2_ = 300 to Z = A = 352 is non heating zone near the exit for inner cylinder while the outer cylinder assumed adiabatic. In radial directions R_i_ = r_i_/b and R_o_ = r_o_/b.

(a) Inner cylinder ($R=R_{i}$):
  *Full Heating*$U=W=0\;\mathrm{and}\;\frac{\partial \theta }{\partial R}=-1\;\mathrm{for}\;0\leq z\leq A$ (heating zone)
  *Partial Heating*$U=W=0\;\mathrm{and}\;\frac{\partial \theta }{\partial R}=-1{\;\mathrm{for}\;Z}_{1}\leq Z\leq Z_{2}$ (heating zone)
  $U=W=0\;\mathrm{and}\;\frac{\partial \theta }{\partial R}=0\;\mathrm{for}\;0\leq Z<Z_{1\;}{\mathrm{and}\;Z}_{2}<Z\leq A$ (non-heating zone)
(b) Outer cylinder ($R=R_{o}$): $U=W=0\;\mathrm{and}\;\frac{\partial \theta }{\partial R}=0\;\mathrm{for}\;0\leq Z\leq A$
(c) Inlet (Z=0): $\frac{\partial U}{\partial Z}=\frac{\partial W}{\partial Z}=0\;\mathrm{and}\;\theta =0{\;\mathrm{for}\;R}_{i}<R<R_{o}$
(d) Outlet (Z=A): $\frac{\partial U}{\partial Z}=\frac{\partial W}{\partial Z}=0\;\mathrm{and}\;\frac{\partial^{2}\theta }{\partial Z^{2}}=0,{\;R}_{i}<R<R_{o}$.

The scheme has a two-step (predictor-corrector algorithm). So in the first steps (predictor step), Equations (2)–(4) are marched advance in time by considering the diffusion terms implicitly to yield provisional estimates of the velocity field $U_{i}^{*}$ and the temperature field $\theta^{n+1}$ at the new time level (n + 1). This is illustrated mathematically, as follows:

(5) $$U_{i}^{*}-\mathrm{Pr}\left[\delta \tau \left(\frac{\partial^{2}U_{i}^{*}}{\partial X_{j}^{2}}\right)\right]=U_{i}^{n}-\;\delta \tau \left(U_{j}^{n}\frac{\partial U_{i}^{n}}{\partial X_{j}}+\;\frac{\partial P^{n}}{\partial X_{i}}-{\mathrm{Ra}\;\mathrm{Pr}\;\theta }^{n}\right)$$

(6) $$\theta^{n+1}-\delta \tau \left(\frac{\partial^{2}\theta^{n+1}}{\partial X_{j}^{2}}\right)=\theta^{n}-\;\delta \tau \left(U_{j}^{n}\frac{\partial \theta^{n}}{\partial X_{j}}\right),$$

where δτ is change in nondimensional time.

In the corrector step, a vorticity preserving and irrotational correction pressure field is used to correct the non-solenoidal provisional approximation for velocity field. The free velocity field of divergence at the latest time level, $U_{i}^{n+1}$ and related pressure field ${\;P}^{n+1}$, is calculated to fulfill the conservation of momentum as follows:

(7) $$U_{i}^{n+1}-\mathrm{Pr}\left[\delta \tau \left(\frac{\partial^{2}U_{i}^{*}}{\partial X_{j}^{2}}\right)\right]=U_{i}^{n}-\;\delta \tau \left(U_{j}^{n}\frac{\partial U_{i}^{n}}{\partial X_{j}}+\;\frac{\partial P^{n+1}}{\partial X_{i}}-{\mathrm{Ra}\;\mathrm{Pr}\;\theta }^{n}\right).$$

Subtracting Equation (5) from Equation (7), we have

(8) $$U_{i}^{n+1}\;-U_{i}^{*}=-\;\delta \tau \frac{\partial }{\partial X_{i}}\left(P^{n+1}-P^{n}\right),$$

where correction of pressure field is expressed as:

(9) $$P^{\prime }=P^{n+1}-P^{n}.$$

Then, Equation (8) is reduced as,

(10) $$U_{i}^{n+1}\;-U_{i}^{*}=-\;\delta \tau \frac{\partial P^{\prime }}{\partial X_{i}}.$$

For making the velocity free from the divergence at new time level, take the divergence of Equation (10), hence,

(11) $$\nabla^{2}P^{\prime }=\;\frac{\partial U_{i}^{*}/\partial X_{i}}{\delta \tau }.$$

So, by solving the Poisson Equation (11) we obtained the correction of the pressure. As suggested by Saad and Schultz [13], Cheng and Armfield [14] and stated in Hasan et al. [15], the Equation (11) is solved for the interior nodes using GMRES solver with the following boundary conditions.

(i) at inflow and solid walls of the annulus: $\frac{\partial P^{\prime }}{\partial n}=\;0$
(ii) at out flow of the annulus: $P^{\prime }=\;0$,

where *n* is normal to the boundary of the domain at any location.

Once by obtaining the pressure correction field, Equations (9) and (10) can be used for the inside the flow domain to correct the pressure and predicted velocity field.

In current time the pressure field can be obtained as:

(12) $$P^{n+1}=P^{n}+P^{\prime }.$$

The corrected velocity field will be recovered at the current time as,

(13) $$U_{i}^{n+1}=U_{i}^{*}-\;\delta \tau \frac{\partial P^{\prime }}{\partial X_{i}}.$$

At the solid walls, for all the velocities no-slip and no penetration condition is specified explicitly. The ordinary momentum equation is used to update the pressure at the inlet boundary and solid walls. To obtain pressure at the outflow, the traction free condition proposed as fallows by Gresho [16] are employed:

(14) $$-P+2\mu \left(\frac{\partial u_{n}}{\partial n}\right)=0.$$

This condition has also been employed by Cheng and Armfield [14] and Hasan et al. [15]. A hybrid scheme is used for discretization of convective terms, which is based on local-cell Peclet number Pe. The scheme applied is either a 4th order accurate central differencing or a 3rd order upwind scheme proposed by Kuwahara [17]. For avoiding the spurious grid-scale pressure oscillations, a divergence operator at cell faces is applying for discretization of correction pressure-Poisson equation as follows. 

The 4th order central-difference scheme employing five grid points is expressed as:

(15) $$U{\frac{\partial U}{\partial R}|}_{i,j}={\;U}_{i,j}\left\{\frac{-U_{i-2,j}+8\left(U_{i+1,j}-U_{i-1,j}\right)\;–{\;U}_{i+2,j}}{12\delta R}\right\}.$$

The 3rd order upwind scheme employing five grid points is expressed as:

(16) $$U{\frac{\partial U}{\partial R}|}_{i,j}={\;A}_{i,j}+B_{i,j},$$

where

(17) $$A_{i,j}\;={\;U}_{i,j}\left\{\frac{-U_{i+2,j}+8\left(U_{i+1,j}-U_{i-1,j}\right)\;-{\;U}_{i-2,j}}{12\delta R}\right\}$$

and

(18) $$B_{i,j}\;=\left|U_{i,j}\right|\left\{\frac{U_{i+2,j}-4U_{i+1,j}+6U_{i,j}\;-\;4U_{i-1,j}+{\;U}_{i-2,j}}{4\delta R}\right\}.$$

The fluctuation-pressure gradient terms are first-order derivatives which are discretized as second-order centrally accurate terms near the wall and fourth-order accurate terms inside the flow. They are respectively expressed as:

(19) $${\frac{\partial P}{\partial R}|}_{i,j}=\;\frac{P_{i+1,j}\;-{\;P}_{i-1,j}}{2\delta R}$$

(20) $${\frac{\partial P}{\partial R}|}_{i,j}=\;\frac{-P_{i-2,j}+8\left(P_{i+1,j}-P_{i-1,j}\right)\;-{\;P}_{i+2,j}}{12\delta R}.$$

For Neumann condition at the boundary, the expression for discrete derivative should be one sided differencing expression for better accuracy. These are as follows:

Forward-difference scheme using three grid points is expressed as:

(21) $${\frac{\partial U}{\partial R}|}_{i,j}=\;\left(\frac{3U_{i,j}\;-\;4U_{i+1,j}\;+{\;U}_{i+2,j}}{4\delta R}\right).$$

Forward-difference scheme employing five grid points is expressed as:

(22) $${\frac{\partial U}{\partial R}|}_{i,j}=\left(\frac{25U_{i,j\;}-\;48U_{i+1,j}\;+\;36U_{i+2,j}\;-\;16U_{i+3,j\;}+\;3U_{i+4,j}}{12\delta R}\right).$$

**Figure 3:**
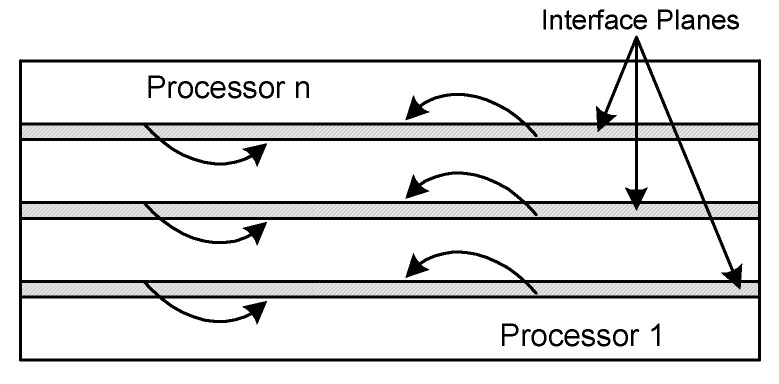
1D “slab” decomposition of a 2D domain.

The parallel code has been developed which runs on distributed memory devices using MPI (Message Passing Interface). The straight-forward method of parallelization is to use the decomposition of the domain. In which the computation is equally split between the n processors. Using the MPI commands like MPI SEND, MPI RECV and MPI WAIT, explicit point-to-point communication is introduced. While in operations for data set at two different parts of the code, it is necessary communication between processors. When derivatives terms are calculated, each processor needs data for the interface neighboring horizontal planes so that each processor exchanges one dimensional data from the other processors. The schematic view of the domain decomposition employed is shown in Figure 3.

**Figure 4:**
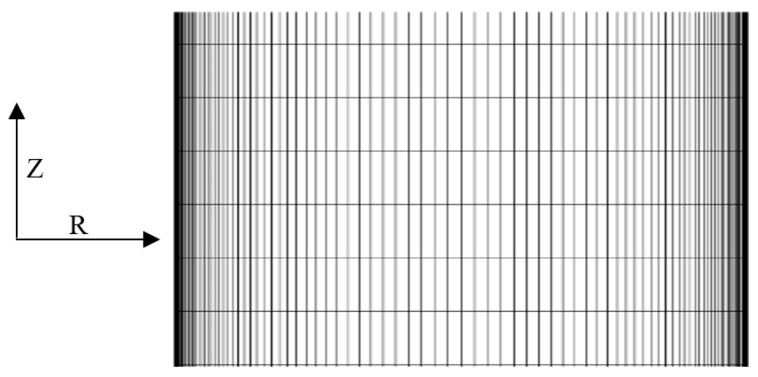
Grid structure of 100 × 2500 for Numerical simulation.

Non-equidistant meshes are used for most near-wall for simulation as shown in Figure 4. A mapping-function is used to mapped the physical space in radial direction (R) into the computational space ε(R). Now seeing the Figure 4, it is clearly shown that a non-equidistant meshes (fine near both wall and coarse in the middle) is used in the radial direction (normal to the wall). And, while a constant mesh is used for axial direction. An asymmetric hyperbolic tangent coordinate is used for refinement the mesh in the r-direction towards the wall, as follows:

(23) $$R\left(\varepsilon \right)=\frac{1}{2}+\frac{1}{2}\left[\frac{\tanh\left\{\delta \left(\varepsilon -\frac{1}{2}\right)\right\}}{\tanh\left(\frac{\delta }{2}\right)}\right],$$

where ε ∈ (0,1) and R ∈ (0,1) and the parameter δ determine the quantity of wall-normal grid point strengthening.

Now for any physical quantity to be solved, which may be either U, W or θ and so forth, the stretching function is introduced. The first derivative of a quantity θ in physical space are determined in the computational space (ε) and subsequently divided with the Jacobian as:

(24) $$\frac{\partial \theta }{\partial R}=\;\frac{1}{\left(\frac{\partial R}{\partial \varepsilon }\right)}\frac{\partial \theta }{\partial \varepsilon },$$

where $\left(\frac{\partial R}{\partial \varepsilon }\right)$ is the Jacobian term.

## 3. Validation of Scheme

**Figure 5:**
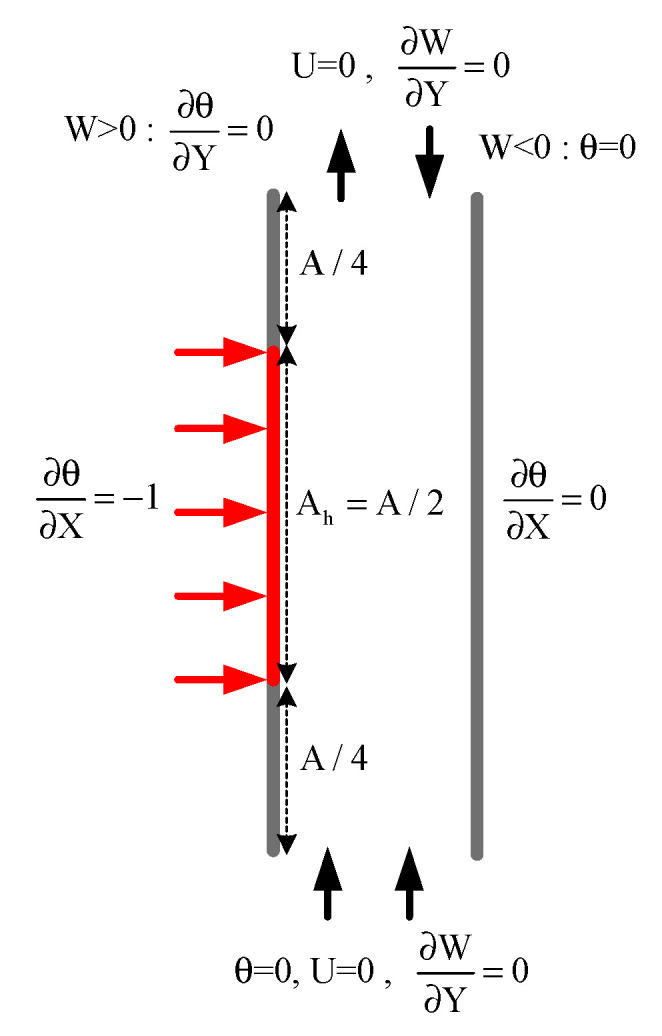
Benchmark configuration with boundary condition.

The numerical scheme used in this analysis has been validated with the data achieved by Desrayaud et al. [7] and Amine et al. [18] for 2-D laminar, steady flow in an open vertical channel, influenced by natural convective heat transfer. The thermally driven flow in vertical channels served as a benchmark problem and Desrayaud et al. [7] have provided a benchmark solution for natural convection flows in vertical channel. They tested with two vertical channels, one partly heated at constant heat flux and other channel kept as insulated for free convective flow of air. The problems of this kind lead to a so-called flow reversal when the one wall of channel are exposed to asymmetric heating. Amine et al. [18] also dealt with air for the natural convection between two vertical walls. The geometrical shape with boundary condition taken for validation are shown in Figure 5. The results of the validation are seen in the paper released by Mustafa et al. [9].

## 4. Results and Discussion

**Figure 6:**
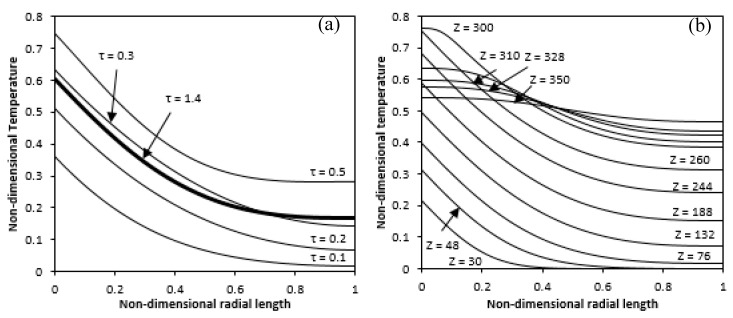
Temperature variation along the radial direction (**a**) at mid-height with time (**b**) at different axial length (Ra = 4.4 × 10^4^).

First, the temperature profile along the radial length in the middle of the annulus is shown for different selected times and at this position, the temperature overshoot can be observed in Figure 6a. As seen, with time reaching a maximum, the temperature at any radial location goes up and then decreases again to a lower value. Interpretation of this behaviour refers to the fact that the quantity of induced flow at the initial time is small; thus, the coefficient of heat transfer would be lower and the diffusion would dominate the convection. As a result, the process of transfer of heat is primarily by conduction because the mechanism of heat removal by axial velocity is not sufficiently effective to provide a heat balance that causes the temperature to rise to a definite value. More heat can be conducted into the annular fluid gap after a while to increase the buoyancy force, which pushes more fluid into the gap. It would result in a higher coefficient of heat transfer; thus, the heat reduction mechanism is strong enough to reduce the temperature. The temperature profile for different axial lengths at a steady state was also plotted along the radial direction in Figure 6b. The dimensional-less temperature in the lower and upper non-heating zones tends to be almost zero and the temperature variation starts from where the heating starts and having a minimum value at the adiabatic wall.

**Figure 7:**
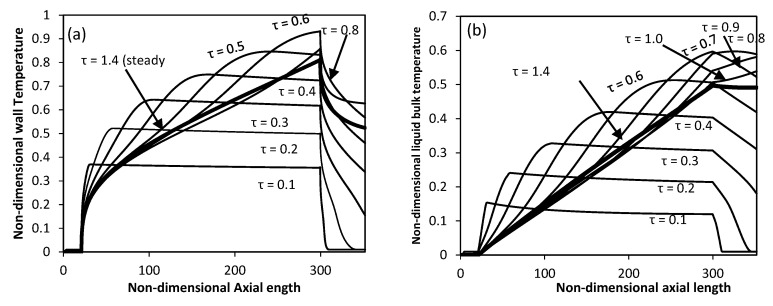
Variation of Temperature along the axial length with time for partial heated inner wall (**a**) wall temperature (**b**) liquid bulk temperature (Ra = 4.4 × 10^4^).

Other results like wall and lquid temperature were also generated to compare the non-dimensional wall variation and mean bulk fluid temperatures along the axial length at selected non-dimensional times are shown in Figure 7a,b. The graphs show the zero value of dimensional temperatures in the non-heating lower zone, for both wall and liquid bulk. The wall temperature descends abruptly for the upper non-heating zone while the temperature of the liquid bulk becomes constant. Both temperatures in the middle heating zone grow linearly. 

**Figure 8:**
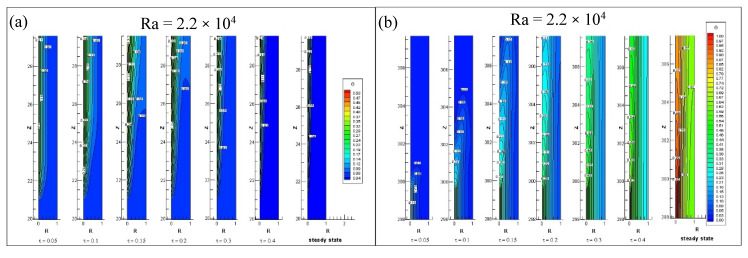
Contours of temperature profile at (**a**) the beginning and (**b**) the end of heating of the annulus.

Figure 8 illustrate the transient rise of the non-dimensional temperature profile at the starting and near exit of annulus inner heated cylindrical wall. The Figure 8a shows thermal boundary layer formation near the beginning of heating starts at lower region of annulus. In time, first the thickness of boundary layer grows perpendicular to the inner wall (in radial direction) but as the time march the induced velocity of flow rises, So the thickness of thermal boundary layer in radial direction decreases until it gets steady sate. The Figure 8b indicates the transient development of the non-dimensional temperature profile near the heating end. As the time runs out, the increase in temperature in radial as well as axial directions. As the Rayleigh increases, the steady state is earlier achieved due to decreases in transient time. 

**Figure 9:**
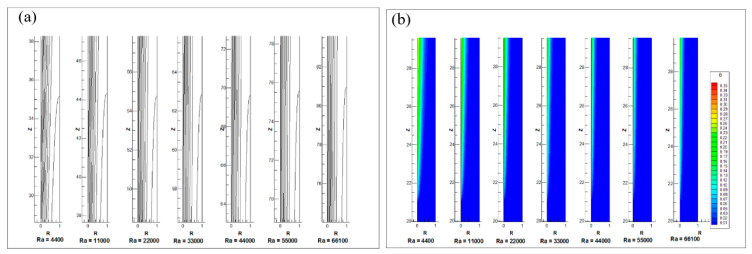
Comparison of (**a**) thermal boundary layer profile and (**b**) temperature profile along the axial length for different Rayleigh numbers.

Figure 9a displays the profile of the boundary layer for the different Rayleigh numbers. It is seen here that the length of the thermal entrance region from which the flow is fully developed increases with an increase in the number of Rayleigh. Figure 9b provides a comparison of contour temperature for different Rayleigh numbers at the lower zone of the annulus. As shown in Figure 9b, the non-dimensional temperature profile near the inside wall is suppressed at the beginning of the heating wall, with Rayleigh number. That is, the transfer of heat by conduction is higher at low Rayleigh number. The transfer of heat from the convection increases with Rayleigh number. Hence the temperature of wall and the thickness of thermal boundary layer decreases.

**Figure 10:**
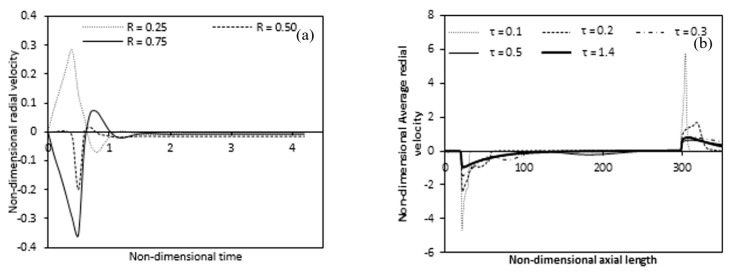
Variation of local and average radial velocity (**a**) at a different radial location (**b**) along the axial direction with time at mid-height (Ra = 4.4 × 10^4^).

The time measurement of radial velocity at mid-height of the annulus for various radial positions is indicated in Figure 10a. As the radial velocity initially fluctuates when heating begins but when the flow is stabilized, the radial velocity is constant over time and nearly zero for different radial locations at mid-height. This means that after some time, the flow is one-dimensional and the radial velocity components vanish, which means that fully developed flow at this location. The transient behavior of the average radial velocity along the axial length shows in Figure 10b. The figure shows the fluctuation accurse in the lower and upper non-heated region and the highest fluctuation accurses near the inner cylinder. A negative average radial velocity appears in the region from where the heating begins at the steady-state and it decreases along the axial length and disappears where the flow becomes fully developed. Once again, the average radial velocity in the upper non-heated region appears with a positive value.

**Figure 11:**
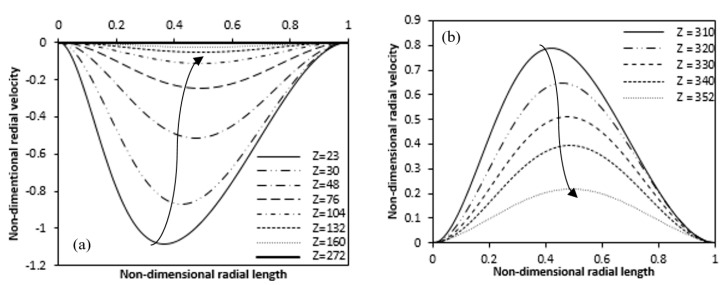
Variation of radial velocities at different axial lengths along the radial direction for (**a**) Heating zone (**b**) Non-heating upper zone. (Ra = 4.4 × 10^4^).

Change in radial velocity along the radial length is presented in Figure 11, for the heating zone at a specific axial position and the upper zone, which is not heated, respectively. The negative radial velocity decreases along the axial length for the unheated lower region while a positive radial velocity decreases in the upper unheated region along the axial length. The radial velocity is nearly zero at the inlet but increases as we move up the fluid toward the heated inner wall. More fluid moves towards the heated inner cylinder due to heating. This is why in Figure 11a, higher negative radial velocity is seen near the beginning of the heated inner wall. Figure 11a also clearly shows that the negative radial velocity decreases and the peaks move from the inner heated wall to the outer wall along the axial length of the heated zone. The radial velocity almost vanishes where the flow is fully developed. From the Figure 11b it is clear that at the end of the heating of the inner wall, the radial velocity becomes positive and high where the heating ends and then it decreases along the axial length in the unheated upper zone.

**Figure 12:**
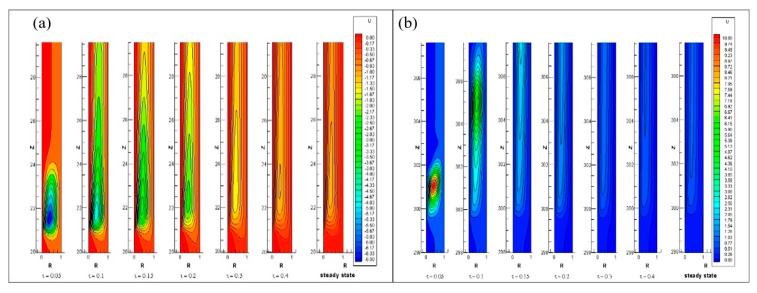
Contours of radial velocity component for Ra = 4.4 × 10^3^ at (**a**) the beginning and (**b**) heating end.

The transient development of radial velocity contours at the inlet as well as exit of heated cylindrical wall are shown in Figure 12. It is shown in Figure 12a, the negative radial velocity contours emerge with Rayleigh numbers in the starting of heated inner cylindrical wall, whereas the contour of positive radial velocity develop in the upper region of the annuli shown in Figure 12b. Over time, at a steady-state, the axial length of all these contours should stretch and maintain a flattened shape.

**Figure 13:**
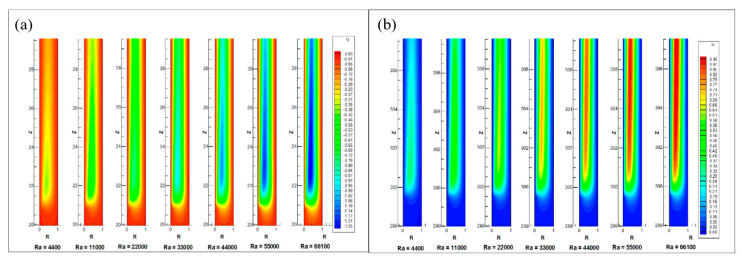
Comparison of radial velocity at (**a**) the beginning and (**b**) end of the inner heating wall of the annulus at different Rayleigh Number.

This also indicates that, for the same Rayleigh number, the width of the contours of radial velocity is decreasing over time. Yet the width of the contours of radial velocity increases as Rayleigh number increases. Near the place where the heating begins, the magnitude of radial velocity formed is negative and its amplitude falls over an axial length, which reach nearly zero value when flow becomes fully developed. Furthermore, positive radial velocity is established near the place where heating stops and its strength falls over the axial length up to the exit. The steady-state contour of radial velocity for different Rayleigh numbers at the beginning and end of the heating zone shown in Figure 13a,b. As the Rayleigh number increase, the negative radial velocity magnitude increases near the starting of the heat, while at the end of the heat, the positive radial velocity magnitude increases.

**Figure 14:**
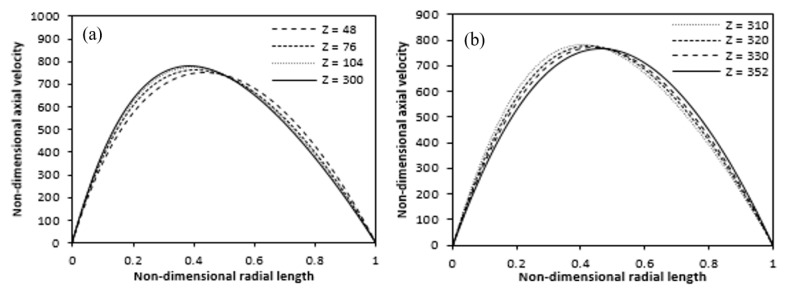
Variation of axial velocity at different axial positions along the radial length (**a**) heating zone (**b**) non-heating upper. (Ra = 4.4 × 10^4^).

The axial velocity variation over the radial length is displayed in Figure 14 for various annulus axial positions. Figure 14a shows that axial velocity peaks in the heated region shift towards the heated wall. And then, Figure 14b suggests that the axial velocity peaks shift towards the outer adiabatic wall in the unheated upper region.

**Figure 15:**
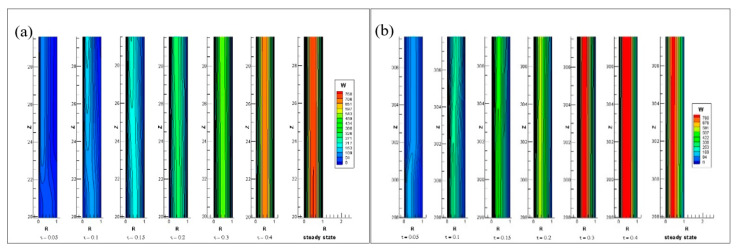
Axial velocity contours at (**a**) the beginning and (**b**) the end of the annulus inner heated wall.

Figure 15a,b represented the transient growth of an axial velocity at the starting and end of the annulus inner heated cylinder respectively. In the beginning, the axial velocity increases in the heated wall region and as time passes, it starts to expand in the entire annulus. By Figure 15 the axial velocity is increasing and its intensity is increasing with time. A similar pattern is also seen here, like that for the axial velocity in the fully heated case, with the Rayleigh number increasing, the magnitude of axial velocity increases.

**Figure 16:**
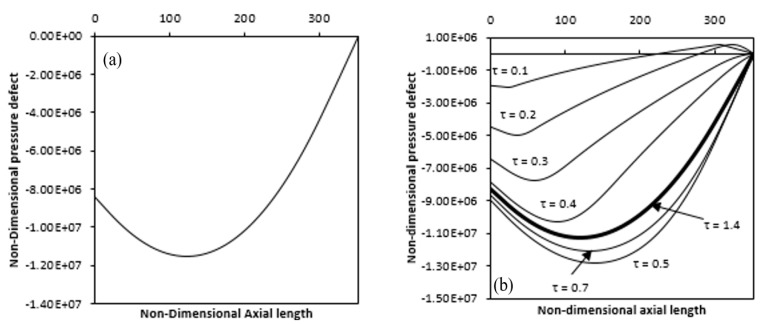
Pressure defect variation along the axial length over time (**a**) fully heated annulus (**b**) partial heated annulus (Ra = 4.4 × 10^4^).

Figure 16a shows a variation in the axial length of the dimensionless pressure defect. The value is negative from the annulus inlet, that drop along the axial direction reaches a minimum value and then rises to the annulus exit. It can also be seen that the dimensionless pressure defect first decreases to a minimum value from the inlet value at the annulus entry and then rises again to zero value at the end of the annulus. Such pressure action is because the induced fluid near the entrance absorbs less heat and thus has a lower magnitude of the force of buoyancy relative to the viscous force (wall and fluid friction + inertia force). Consequently, the viscous force overcomes the force of the buoyancy, so that the pressure defect decreases along the axial length. As the fluid moves up through the annulus, it consumes more heat and raises the buoyancy force until it becomes equivalent to the frictional force where the pressure becomes minimal. When the fluid continues to travel upward, absorbing still more heat, the force of the buoyancy enhances.

So as to overcome the viscous force, resulting in an increase of the pressure defect. Figure 16b indicates a time difference in the pressure defect along the axial length. As time passes, a high magnitude of distribution of the pressure defect along the annulus is obtained, which is similar to those shown in the figure. It is shown that the negative value of the dimensionless pressure defect with time first increases at any position on the axial range, reaches the minima and then decreases to reach some lower value at the steady-state.

This phenomenon is due to the overshooting of temperature, as described earlier. It is shown that in the case of partial annulus heating, the pressure defect is initially positive near the annulus exit (which is the non-heating zone) and subsequently becomes negative.

### Effect of Partial Heating on Different Parameter

**Figure 17:**
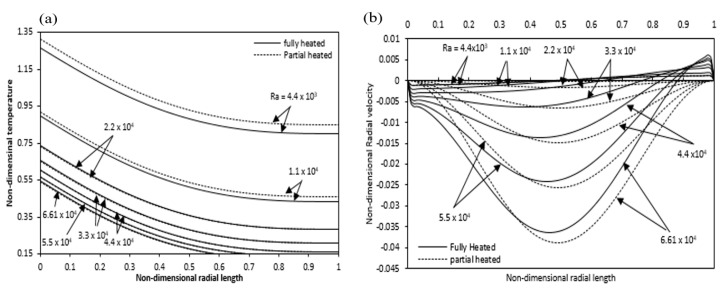
Comparison of (**a**) temperature (**b**) Radial velocity variations at mid-height along the radial direction for partial and full heated annulus for different Ra.

Figure 17a provides a comparison between partial and full heating in which different Rayleigh numbers show temperature difference along the radial direction at annulus mid-height. When we increase the Rayleigh number, the non-dimensional wall temperature decreases at any particular radial location. The non-dimensional temperature increases due to partial heating compared with the fully heated condition, which is more noticeable at low Rayleigh numbers. That is because, in the case of partial heating, the liquid mass flow rate decreased. It is clear that the mass flow rate would be lower at low Rayleigh numbers compared with that at high Rayleigh numbers. Figure 17b indicates the impact of partial heating on the radial velocity at the annulus mid-height. The radial velocity is nearly zero at low Rayleigh number or vanishes at mid-height of annulus, as increase in Rayleigh number, radial velocity increases in negative magnitude. For partial heating case, the negative velocity magnitude is greater than the fully heated case and the peak of negative radial velocity for a partial heating case is moved away from the heated inner cylinder as compared to the fully heated.

**Figure 18:**
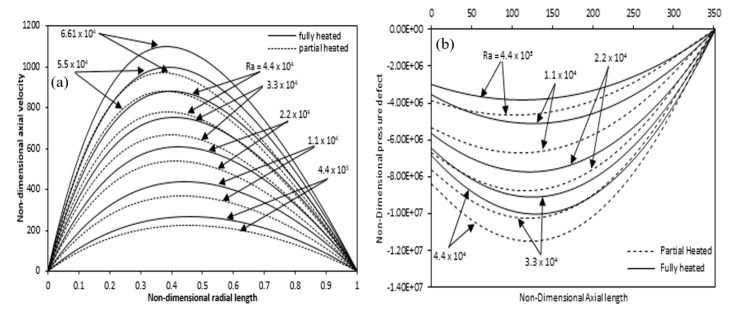
Comparison of (**a**) axial velocity variation at mid-height along the radial length (**b**) pressure defect variation along the axial length for partial and full heated annulus for different Ra.

Figure 18a shows a comparison of axial velocity at various Rayleigh numbers for fully and partially heated annulus. In the case of partial heating, the axial velocity decreases as compared with the fully heated annulus due to the non-heating zones at the beginning and exit of the annulus. For the full and partial heating cases, the difference in the axial velocity variation decreases with Rayleigh number. Figure 18b indicates the pressure defect variation along the axial direction of the annulus. Here also, it is seen that the difference between the full and partial heating pressure defect increases with Rayleigh number. 

The phenomenon of transfer of heat has been characterized as for the average and local Nusselt number, that are estimated at the heated region of the inner wall. The local Nusselt number (Nu) for the inner heated cylinder is defined as:

(25) $$\mathrm{Local}\;\mathrm{Nusselt}\;\mathrm{number},\;{\mathrm{Nu}}_{z}\;=\;\frac{q\;b}{k\left(T_{w,z}-T_{b}\right)}=\;\frac{1}{\left(\theta_{w,z}-\theta_{b}\right)}$$

(26) $${\;T}_{b}\left(\mathrm{dimensional}\;\mathrm{bulk}\;\mathrm{liquid}\;\mathrm{temperature}\right)=\frac{\int^{\;}{\rho C}_{p}\mathrm{wTdA}}{\int^{\;}{\rho C}_{p}\mathrm{wdA}}$$

(27) $$\mathrm{Average}\;\mathrm{Nusselt}\;\mathrm{number}\;\bar{\mathrm{Nu}}=\frac{1}{\left(z_{2}-z_{1}\right)}\int_{z_{1}}^{z_{2}}{\mathrm{Nu}}_{z}\;\mathrm{dz}.$$

**Figure 19:**
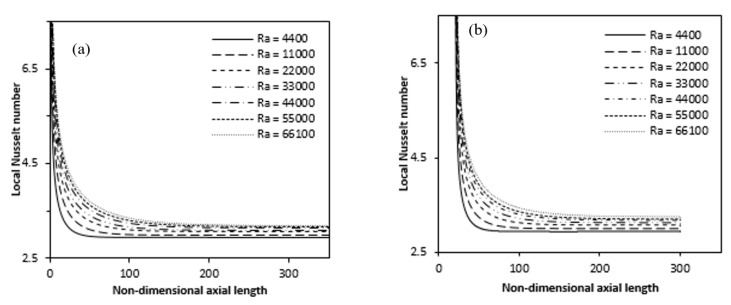
Variation of Local Nusselt number for different Ra over the axial length (**a**) full (**b**) partially heated.

Figure 19a,b show the local Nusselt number variation for full and partially heated cases with the Rayleigh numbers along the heated axial length, respectively. The value of the local Nusselt number sharply decrease from a very large values at the annulus inlet and after gradually becomes constant in the rest of the annulus axial length.

**Figure 20:**
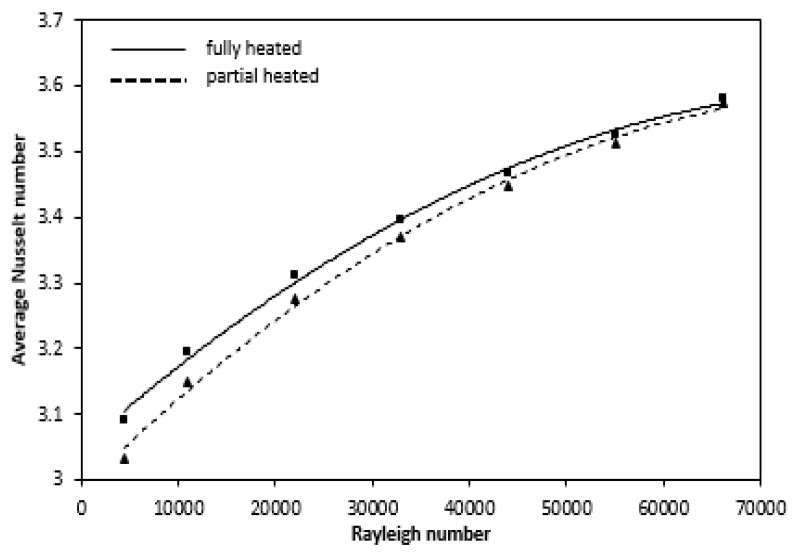
Variation of average Nusselt number at different Ra for the fully and partially heated annulus.

Thus, such variations in the Nusselt number show the development of boundary layer at the annulus entrance, while for the rest of the annulus length the flow is fully developed. The greater the Rayleigh number, increases the Nusselt number for both fully and partially heated cases. Figure 20 represent with Rayleigh number, the variance of the average Nusselt number increases gradually and can be illustrated by the following empirical correlations derived from the curve, as indicate in Table 2. The magnitude of the average Nusselt number decreases due to partial heating but follows the same pattern. From the Figure, the difference in Averaged Nusselt number is more at lower Rayleigh number while these gradually decreases with higher Rayleigh number. The effect of partial heating is more pronounce for low Rayleigh number while for higher Rayleigh number its effect on average Nusselt number is less. 

The percentage mean deviation in each case for n number of data points were evaluated using the following relation:

(28) $$\mathrm{Mean}\;\mathrm{Deviation}\;\left(\mathrm{MD}\right)=\left[\frac{1}{n}\sum_{i=1}^{n}\mathrm{Abs}\left[\frac{\left({\mathrm{Nu}}_{i,p}-{\mathrm{Nu}}_{i,c}\right)}{{\mathrm{Nu}}_{i,c}}\right]\times 100\right],$$

where subscript ‘p’ is for predicted and ‘c’ for calculated value.

The non-dimensional volume flow rate $\bar{Q}$ is expressed through the annulus as:

(29) $$\bar{Q}=\;\int_{R_{i}}^{R_{o}}2\;\pi \;R\;W\;\mathrm{dR}.$$

Similarly, mean bulk axial velocity is define as:

(30) $$\bar{W}=\;\frac{\bar{Q}}{\int_{R_{i}}^{R_{o}}2\;\pi \;R\;\mathrm{dR}}.$$

**Figure 21:**
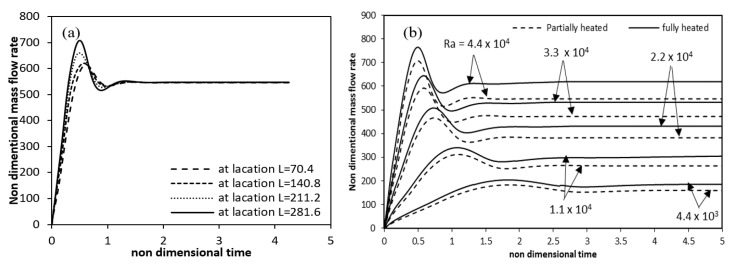
Variation of mass flow rate with time (**a**) at different axial locations (**b**) for fully and partial hated annulus for different Rayleigh numbers.

Figure 21a shows the time variation of dimensionless mass flow rate at different axial locations. From the figures it can be seen that, the mass flow rate at first increases with time, attains a maxima and then decreases to become constant at the steady state condition. At steady state the mass flow rate is almost same at all positions in the axial direction, thereby showing the conservation of mass. Figure 21b provides a comparison of the mass flow rates over time for full and partial heating cases. For the same Rayleigh number, the mass flow rate of partial heating having lower value as compared with the full heating case. As the Rayleigh number increases, this difference increases. 

However, the numerical value of the pressure defect gets calculated. The gradient of the difference between two pressures, the actual static pressure p at any location and the hydrostatic pressure p_h_ which will be at the same point in the absence of any motion that would result in the departure of the pressure field from the hydrostatic variation imposed due to gravity by Gebhart et al. [19]. With buoyancy force and motion, the difference between these two (p − p_h)_, is the pressure change that arises through fluid motion. It is due to acceleration, viscous force and buoyancy force. The difference (p − p_h)_ is called the ‘motion’ pressure field or ‘pressure defect’ by Mohanty and Dubey [20]_._ That is the actual static pressure p is decomposed into p_h_ and p_m,_ as

p = p_h_ + p_m_ (Differential pressure = Hydrostatic pressure + Pressure defect).

**Figure 22:**
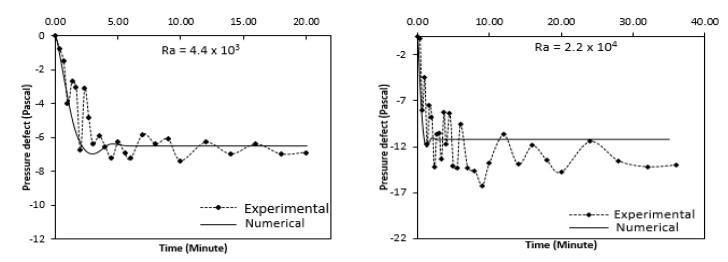

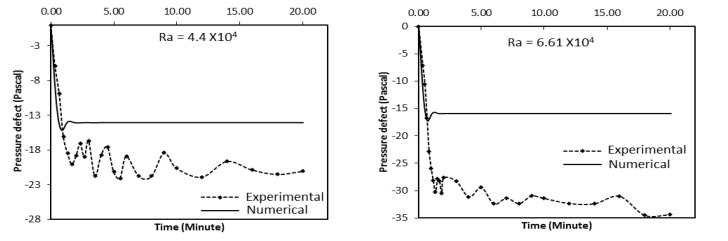
Comparison of the Experimental and Numerical pressure defect of the annulus with time.

The experimental and Numerical values of the pressure defect thus obtained are shown in Figure 22 for different Raleigh number, over time. The numerical values of the pressure defect are seen to initially decrease suddenly and then increase to become constant thereafter, while the experimental values of the pressure defect display almost identical patterns but with great fluctuations. Almost similar trend is seen in the experimental values of the pressure defect but with large fluctuations. At lower Raleigh number, the experimental and the numerical value match well. But with higher Raleigh number, a large deviation is observed. In the real system, as found experimentally, there is large decrease in the pressure defect, especially at higher value of Raleigh number because the friction and bend losses then become more prominent which are not considered in the numerical analysis.

Table 3 display the comparative comparison of thermal entrance length and the average Nusselt number with Rayleigh numbers, respectively, for full and partial heated cases. These parameters have already been discussed in the earlier section.

## 5. Conclusions

Numerical investigations have been conducted for different Rayleigh numbers (Ra = 4.4 × 10^3^ to 6.61 × 10^4^). The study results show the rapid increase in the temperature for any radial location due to conduction having a maxima. As the convection becomes significant, the axial flow is developed and a steady state is observed below the maxima. The flow becomes parallel and fully developed with this eventual steady state which is known as temperature overshoot. The above phenomenon was observed for all Raleigh number. In addition, the convective radial velocity is getting higher by increasing Rayleigh number resulting in a negative effect on the fully developed region and positive effect on the thermal entrance length. The time taken to achieve equilibrium decreased with Rayleigh number due to the positive effect of this number on the buoyancy force. The numerically determined Nusselt number at steady state for fully and partially heated cases comes out to be 3.09 to 3.58 and 3.03 to 3.57, respectively, for Rayleigh number, Ra is 4.4 × 10^3^ to 4.4 × 10^4^. At the beginning of heated wall, local heat transfer coefficient and Nusselt number decrease from very large values and then for the remaining length of annulus it gradually decreases and become constant. The variations in the Nusselt number along the annulus height represent the developing boundary layer at the entrance and fully developed flow in the remaining length. The average Nusselt number increases gradually with Rayleigh number and can be represented in terms of Rayleigh number by the following correlations.

Nu_a_ = 3.049 + 0.00001 Ra − 8 × 10^−11^ Ra^2^ Mean deviation = ±3.15% (Fully Heated)

Nu_a_ = 2.986 + 0.00001 Ra − 9 × 10^−11^ Ra^2^ Mean deviation = ±3.28% (Partially Heated)

The fully developed region decreases as there is an increase in Rayleigh while the thermal entrance length increases. Due to the non-heating zones at inlet and outlet of the annulus, the axial velocity decreases as compared to when the annulus is fully heated. The difference in axial velocity between the fully heated and partially heated annulus increases with increase in Rayleigh number. As the fluid gets heated up, the buoyancy force increases upward in the annulus and the viscous force decreases simultaneously. This is why the Pressure defect first decreases from the inlet value at the entrance, reaches minima where the two forces become equal and then increases again to attain value zero at the annulus exit when the buoyancy force overcomes the viscous force. Finally, the mass flow rate has been shown to increase initially and attain maxima and then decrease to a constant value at the steady sate. For the same Rayleigh number, the mass flow rate decreases in case of partial heating as compared to those in the fully heated case.

## Figures and Tables

**Table 1:** Variables used for the equations.

b	Annular space (m)	L	Length of annulus (m)
T_a_	Ambient temperature (°C)	g	Gravitational force (m · s^−2^)
k	Thermal conductivity (W · m^−1^ · °C^−1^)	q	Heat flux (W/m^2^)
r, R	Dimensional (m) and Non-dimensional Radial distance	α	Thermal diffusion coefficient (m^2^/s)
β	expansion coefficient (K^−1^)	μ	Dynamic viscosity (N · s/m^2^)
ν	Kinematic Viscosity (m^2^ · s^−1^)	C_p_	=Specific heat (J · kg^−1^ · °C^−1^)
u, U	Dimensional and Non-dimensional radial velocity	w, W	Dimensional and Non-dimensional axial velocity
z, Z	Dimensional (m) and Non-dimensional axial distance	T, θ	Dimensional and Non-dimensional temperature
p, P	Dimensional and Non-dimensional pressure	Nu	Nusselt Number (hb/k)
A	Aspect ratio (l⁄b)	t, τ	Dimensional and Non-dimensional time
∂	discrete	δ	change
ε	computational space	n	Time level
*	Predicted value	w	Wall
i	Inner	o	Outer
q,q_w_	Heat flux (W/m^2^)	∈	iteration-error at current time-level
ρ	Density (kg · m^−3^)	i, j	i-th and j-th coordinate direction
RR	Radius ratio	l	liquid

**Table 2:** Empirical correlations of Average Nusselt number.

	Nu_a_	Mean Deviation
Fully heated	3.049 + 0.00001Ra − 8 × 10^−11^ Ra^2^	±3.15%
Partially heated	2.986 + 0.00001Ra − 9 × 10^−11^ Ra^2^	±3.28%

**Table 3:** Comparison of Thermal entrance length, average Nusselt number and axial bulk velocity for fully and partially heated case.

Ra	Thermal Entrance Length (Dimensionless)		Average Nusselt Number	
	Fully Heated	Partially Heated	Fully Heated	Partially Heated
4.4 × 10^3^	16.75	14	3.09	3.03
1.1 × 10^4^	27	23	3.19	3.14
2.2 × 10^4^	37.25	33.5	3.31	3.27
3.3 × 10^4^	45.85	41.5	3.39	3.37
4.4 × 10^4^	53.65	48.5	3.46	3.44
5.5 × 10^4^	60.75	54.5	3.53	3.51
6.61 × 10^4^	66.65	60	3.58	3.57
